# Development and preliminary psychometric evaluation of the vaginal douching practice scale

**DOI:** 10.3389/fgwh.2026.1776421

**Published:** 2026-08-20

**Authors:** İmren Arpacı Kızıldağ, Saliha Yurtçiçek Eren, Feride Yiğit

**Affiliations:** 1 Gaziantep Şehitkamil District Health Directorate, Gaziantep, Türkiye; 2 Department of Midwifery, Faculty of Health Sciences, Muş Alparslan University, Muş, Türkiye; 3 Department of Midwifery, Faculty of Health Sciences, Istanbul Topkapi Universitesi, Istanbul, Türkiye

**Keywords:** reliability, scale development, vaginal douching, vaginal douching practice scale, validity

## Abstract

**Background:**

Vaginal douching remains common in several settings despite its association with adverse reproductive health outcomes. A standardized instrument for assessing how frequently women perform vaginal douching in specific circumstances is lacking.

**Objective:**

To develop the Vaginal Douching Practice Scale (VDPS) and examine preliminary evidence for its content validity, factor structure, and reliability among married women.

**Method:**

This methodological study included 423 married women aged 18 years and older. An initial 17-item pool was generated from the literature, reviewed for content, and pilot-tested with 50 women. Exploratory factor analysis (EFA) and confirmatory factor analysis (CFA) were conducted sequentially in the same sample; therefore, the CFA was treated as an internal model check rather than an independent confirmation.

**Results:**

The mean age of participants was 33.34 ± 8.55 years. The Kaiser-Meyer-Olkin value was 0.959, and Bartlett's test of sphericity was significant (*χ*2 = 8,750.245, *p* < 0.001). EFA yielded a one-factor solution explaining 71.89% of the variance, with loadings ranging from 0.694 to 0.920. Internal consistency was very high (Cronbach's alpha = 0.97), split-half reliability was 0.93, and the two-week test-retest correlation in 31 participants was 0.92. CFA loadings ranged from 0.642 to 0.937; however, global model fit was inadequate (*χ*^2^/df = 4.428, GFI = 0.786, RMSEA = 0.123, 90% CI = 0.116–0.131) despite several incremental indices exceeding 0.90. Composite reliability was 0.975 and average variance extracted was 0.694, but these values do not compensate for the poor global fit.

**Conclusion:**

The VDPS showed strong internal consistency and promising exploratory evidence; however, the proposed one-factor structure was not adequately supported by CFA. The instrument should be regarded as preliminary and requires item refinement and independent validation before routine clinical or research use.

## Introduction

Vaginal douching is traditionally defined as the practice of cleansing the vaginal canal with a liquid solution delivered manually or with the assistance of various devices (1). Although it was historically considered a method of contraception, scientific evidence has demonstrated that vaginal douching does not prevent pregnancy. Despite the lack of medical benefit, the practice remains widespread across many cultures and is commonly performed for reasons such as hygiene, odor control, cultural expectations, and misconceptions about reproductive health (2).

The vaginal environment maintains a naturally acidic pH, typically ranging from 3.8 to 4.5, which plays a crucial role in regulating microbial balance and protecting against infections. Disruption of this environment through practices such as vaginal douching may increase the risk of adverse reproductive health outcomes, including sexually transmitted infections, alterations in the vaginal microbiota, infertility, ectopic pregnancy, and preterm birth (3–6).

The inclusion of only married women in this study was based on both methodological and sociocultural considerations. Previous research has shown that vaginal douching is closely associated with sexual activity, post-coital hygiene practices, and partner-related expectations, and is therefore more prevalent among married and sexually active women (7, 8). Furthermore, guidelines from international health organizations emphasize that vaginal douching behaviors are strongly influenced by cultural norms, hygiene perceptions, and sexual health beliefs. In scale development studies, the use of a relatively homogeneous sample is recommended to improve the accuracy of construct measurement and strengthen internal validity (9). Therefore, restricting the sample to married women allowed for a more consistent evaluation of the targeted construct.

Previous research has typically assessed vaginal douching using single questions or study-specific checklists. These approaches may identify whether a woman has ever douched, but they do not provide a standardized summary of how frequently the practice occurs across sexual, menstrual, symptom-related, hygiene, religious, and healthcare contexts. Rigorous scale development begins with a clearly bounded construct and transparent documentation of item generation, content evaluation, pretesting, factor analysis, and reliability assessment (10–15).

In this study, the target construct was defined as the self-reported frequency of vaginal douching across specified situations. The instrument was not designed to directly measure evaluative attitudes, approval, intentions, or knowledge. The study therefore aimed to develop the Vaginal Douching Practice Scale (VDPS) and evaluate preliminary evidence for its content validity, internal structure, and reliability among married women attending family health centers in Eastern Anatolia.

## Methods

### Study design

This study was conducted using a methodological research design, appropriate for the development and psychometric evaluation of a measurement instrument. The process included item generation based on the literature, expert evaluation for content validity, pilot testing to assess clarity and feasibility, and comprehensive statistical analyses to evaluate validity and reliability.

### Conceptual definition of the construct

The VDPS was designed to measure behavioral frequency: how often a woman reports performing vaginal douching in each of the 17 specific circumstances. The item content covers sexual activity, menstruation, vaginal symptoms, perceived prevention of infection/cancer/pregnancy, toileting, hygiene and well-being, religious worship, and gynecological examinations. These categories were used to ensure content coverage and were not prespecified as separate psychometric subscales.

### Population and sample

The study population consisted of married women aged 18 years and older who attended family health centers in a province in Eastern Anatolia. The sample was selected using a convenience sampling method, and a total of 423 women participated in the study.

In scale development studies, it is generally recommended that the sample size be at least 5–10 times the number of scale items. The initial scale included 17 items; therefore, the final sample exceeded commonly cited respondent-to-item recommendations and the absolute sample sizes generally considered adequate for factor analysis (9, 16–26).

### Eligibility criteria

Women were eligible if they were aged 18 years or older, married, able to understand and complete the questionnaire, and willing to provide written informed consent. Incomplete forms were excluded.

### Scale development process

#### Literature review and item generation

A focused literature review was conducted to identify the reported reasons, contexts, and determinants of vaginal douching practices and to guide the development of the initial item pool. The review was performed in PubMed/MEDLINE, Scopus, Web of Science, CINAHL, Google Scholar, Turkish national databases, and relevant thesis databases for studies published between 2012 and 2022. The search terms included combinations of the following keywords: “vaginal douching,” “vaginal douche,” “intravaginal practices,” “vaginal hygiene,” “genital hygiene,” “women,” “reproductive health,” “sexual health,” “scale development,” “questionnaire,” and their Turkish equivalents, including “vajinal duş,” “vajinal yıkama,” “genital hijyen,” “intravajinal uygulamalar,” “kadın sağlığı,” and “ölçek geliştirme.”

Studies were considered relevant if they reported vaginal douching behaviors, reasons for douching, cultural or religious determinants, hygiene-related motivations, post-coital or post-menstrual practices, symptom-related douching, or misconceptions related to pregnancy prevention, sexually transmitted infections, or cervical cancer. Review articles, methodological papers on scale development, and national studies addressing genital hygiene practices were also examined to support conceptual and methodological development.

Based on this review, the main content domains were identified as sexual activity, menstruation, vaginal symptoms, perceived prevention of infection/cancer/pregnancy, toileting, hygiene and well-being, religious worship, and gynecological examinations. These domains were used only to ensure content coverage during item generation and were not prespecified as separate psychometric subscales. An initial pool of 17 items was generated, with each item beginning with “I perform vaginal douching…”. Response options were standardized to assess frequency rather than attitudes.

#### Expert review and content validity

The items were evaluated using the Lawshe method by an expert panel of seven academics. The panel included experts in obstetrics and gynecology nursing and consisted of professors, associate professors, and assistant professors. Experts rated each item as “remove,” “revise,” or “retain”. The critical content validity ratio (CVR) was 0.62. All 17 items met the prespecified threshold, no item was eliminated, and wording changes were made in response to expert recommendations.

#### Pilot testing

The revised 17-item form was pilot-tested with 50 women from the target population to assess clarity, comprehensibility, response-option suitability, and feasibility. No items were removed after pilot testing; minor wording refinements were incorporated before the main study. Pilot participants were not included in the main psychometric sample.

### Data collection tools

#### Personal information form

This form consisted of nine items assessing participants' demographic and reproductive characteristics, including age, education, employment status, income level, chronic disease status, number of pregnancies, and number of children.

#### Vaginal douching practice scale (VDPS)

The VDPS consists of 17 items scored on a five-point frequency scale: 1 = never, 2 = rarely, 3 = sometimes, 4 = often, and 5 = always. No item is reverse-scored. Item scores are summed to obtain a total score from 17 to 85; higher scores indicate more frequent self-reported vaginal douching across the situations represented by the scale. No diagnostic or risk cutoff has been established, and scores should be interpreted as continuous. The scale does not directly measure attitudes, beliefs, approval, knowledge, or intentions.

#### Data collection

Data were collected by face-to-face interviews between June 8, 2023, and December 1, 2023. Participants were informed about the purpose of the study, confidentiality, and voluntary participation. Completion took approximately 15 min. For temporal stability, the VDPS was re-administered to 31 participants after two weeks.

#### Ethical considerations

Ethical approval was obtained from the Muş Alparslan University Scientific Research and Public Ethics Committee (May 22, 2023; Decision No: E-10879717-050.01.04-93443). Institutional permissions were secured prior to data collection. All participants provided written informed consent. The study was conducted in accordance with the principles of the Declaration of Helsinki, ensuring confidentiality and voluntary participation.

#### Statistical analysis

Data were analyzed using SPSS version 24.0. Descriptive statistics (percentages, means, and standard deviations) were used to summarize participant characteristics.

For EFA, sample adequacy was examined using the Kaiser-Meyer-Olkin statistic and Bartlett's test of sphericity. Factors were retained using eigenvalues greater than 1, scree-plot inspection, and theoretical interpretability. The extraction method was principal component analysis (PCA). Because a single factor was retained, rotation was not required. Corrected item-total correlations of at least 0.30 were considered acceptable.

CFA was performed using IBM SPSS AMOS version 24.0. EFA and CFA were conducted sequentially using the same 423 participants. Consequently, CFA was interpreted as an internal model check and not as independent confirmation. Model fit was evaluated using *χ*2/df, CFI, TLI, IFI, NFI, GFI, and RMSEA with its 90% confidence interval. Common benchmarks were treated as guides rather than absolute decision rules: values near or above 0.90 for incremental indices indicate minimally acceptable fit, and RMSEA values below approximately 0.06 indicate good fit (10, 12, 14).

Internal consistency was evaluated using Cronbach's alpha, corrected item-total correlations, and split-half reliability. Test-retest stability was summarized using the correlation between the two administrations. Composite reliability (CR) and average variance extracted (AVE) were calculated from the standardized CFA loadings. Because no established comparator instrument was administered, criterion and convergent validity against external measures could not be evaluated. In a one-factor model, discriminant validity between latent dimensions is not applicable.

McDonald's omega total was calculated from the raw item-level data using a one-factor common-factor model. A bootstrap 95% confidence interval was estimated for the omega coefficient.

## Results

### Participant characteristics

The mean age of the participants was 33.34 ± 8.55 years (range, 19–74). More than half were unemployed (56.7%), 40.0% had a university education, and 78.5% reported moderate income. Most of the participants reported one to four pregnancies (76.1%), one to four births (74.7% of valid responses), and one to four children (75.4%) (Table 1). The KMO and Bartlett's test results are presented in Table 2.

**Table 1:** Sociodemographic and reproductive characteristics of participants.

Variable	*N*	%
Age, years (min–max: 19–74) (Mean ± SD:33.34 ± 8.55)		
18–24	43	10.2
25–34	222	52.5
35–44	113	26.7
≥45	45	10.6
Education		
Primary school or below	123	29.1
High school	111	26.2
University	169	40.0
Master's/Doctorate	20	4.7
Employment		
Employed	183	43.3
Unemployed	240	56.7
Income		
Poor	74	17.5
Moderate	332	78.5
Good	17	4.0
Chronic disease		
Yes	68	16.1
No	355	83.9
Pregnancies		
None	45	10.6
1–4	322	76.1
≥5	56	13.3
Births*		
None	69	16.3
1–4	316	74.7
≥5	37	8.8
Children		
None	69	16.3
1–4	319	75.4
≥5	35	8.2

* Valid *n* = 422 for number of births; one response was missing.

**Table 2:** KMO and Bartlett's test values for the vaginal douching practice scale.

Statistic	Value
Kaiser-Meyer-Olkin (KMO) Measure	0.959
Bartlett's Test *χ*^2^	8,750.245
Degrees of freedom	136
*p* value	<0.001

### Content validity and pilot testing

Content validity was evaluated using the Lawshe technique with seven experts. The critical CVR value was accepted as 0.62. All 17 items met the minimum CVR criterion. Item-level CVR values ranged from 0.714 to 1.000. The mean CVR was 0.815, S-CVI/Ave was 0.908, and S-CVI/UA was 0.353. Therefore, no item was removed based on expert content-validity evaluation.

### Item analysis and reliability

Corrected item-total correlations ranged from 0.482 to 0.847, exceeding the 0.30 criterion for all items (Table 3). Cronbach's alpha was 0.97, and alpha remained 0.97 when any single item was deleted. Split-half reliability was 0.93. The two-week test-retest correlation was 0.92 in 31 participants. Although these findings indicate strong score consistency, the very high alpha and multiple high item-total correlations may reflect overlapping item content rather than uniformly greater measurement quality. McDonald's omega total was 0.976, with a bootstrap 95% confidence interval of 0.972–0.979, indicating high internal consistency.

**Table 3:** Corrected item–total correlations and Cronbach's alpha if item deleted.

Scale items	Corrected item–total correlation	Cronbach's alpha if item deleted
1. I perform vaginal douching before sexual intercourse	0.576	0.97
2. I perform vaginal douching after sexual intercourse.	0.725	0.97
3. I perform vaginal douching when I feel my menstrual period is about to start.	0.482	0.97
4. I perform vaginal douching after my menstrual period ends.	0.781	0.97
5. I perform vaginal douching when I have vaginal discharge.	0.719	0.97
6. I perform vaginal douching when I experience itching.	0.731	0.97
7. I perform vaginal douching to protect myself from sexually transmitted infections.	0.736	0.97
8. I perform vaginal douching to protect myself from cervical cancer.	0.667	0.97
9. I perform vaginal douching to prevent pregnancy.	0.504	0.97
10. I perform vaginal douching after urination.	0.710	0.97
11. I perform vaginal douching after a bowel movement (defecation).	0.727	0.97
12. I perform vaginal douching to avoid smelling bad.	0.847	0.97
13. I perform vaginal douching to feel clean.	0.841	0.97
14. I perform vaginal douching to feel good.	0.793	0.97
15. I perform vaginal douching before religious worship (e.g., before prayer/salah).	0.799	0.97
16. I perform vaginal douching before a gynecological examination.	0.784	0.97
17. I perform vaginal douching after a gynecological examination.	0.800	0.97

### Exploratory factor analysis

**Figure 1:**
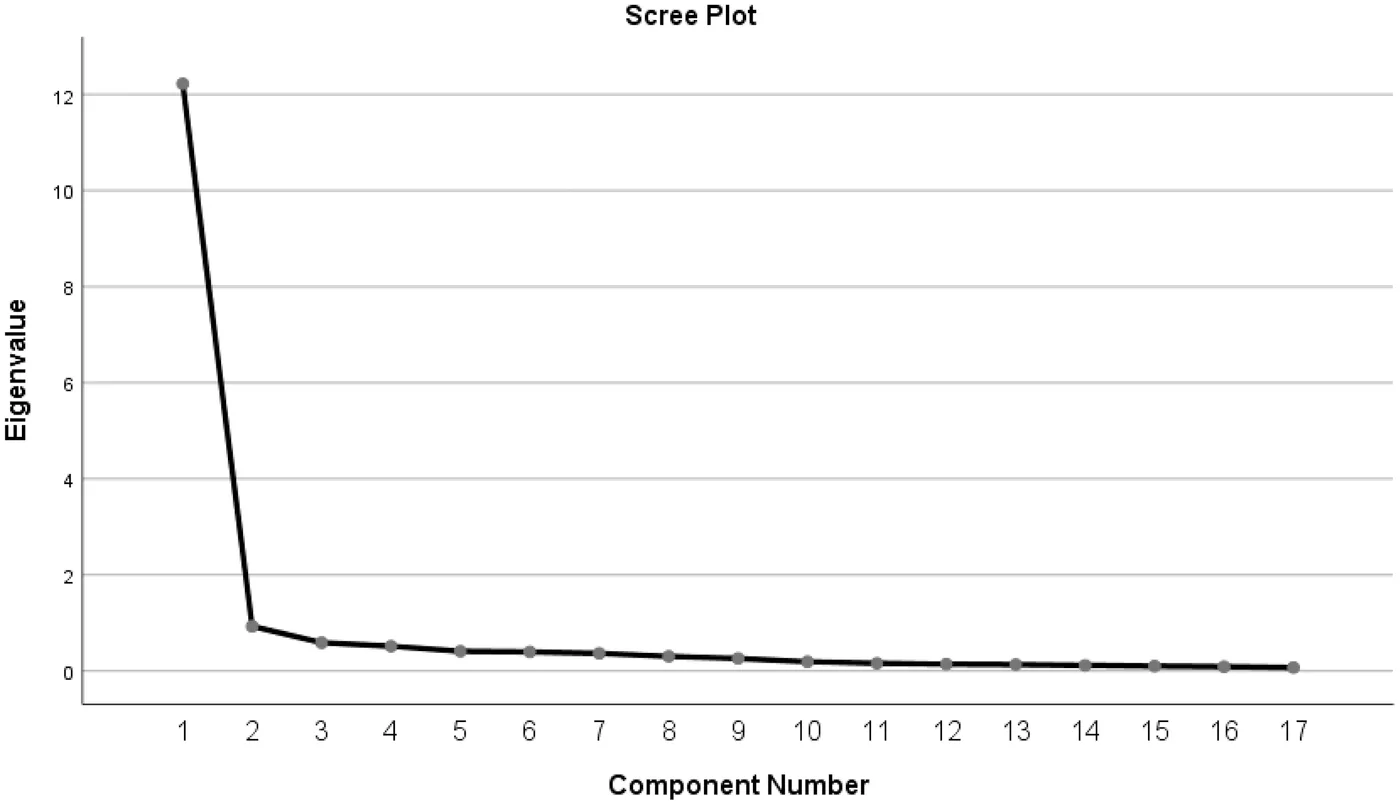
Scree plot from the exploratory factor analysis. The pronounced break after the first component is consistent with a dominant first factor.

The KMO value was 0.959 and Bartlett's Test of Sphericity was significant (*χ*^2^ = 8,750.245, df = 136, *p* < 0.001), supporting the suitability of the correlation matrix for factor analysis. EFA produced a one-factor solution with an eigenvalue of 12.222, accounting for 71.89% of total variance. Factor loadings ranged from 0.694 to 0.920 (Table 4 and Figure 1).

**Table 4:** Exploratory factor analysis loadings.

Item	Factor loading
1. I perform vaginal douching before sexual intercourse	0.759
2. I perform vaginal douching after sexual intercourse.	0.851
3. I perform vaginal douching when I feel my menstrual period is about to start.	0.694
4. I perform vaginal douching after my menstrual period ends.	0.884
5. I perform vaginal douching when I have vaginal discharge.	0.848
6. I perform vaginal douching when I experience itching.	0.855
7. I perform vaginal douching to protect myself from sexually transmitted infections.	0.858
8. I perform vaginal douching to protect myself from cervical cancer.	0.817
9. I perform vaginal douching to prevent pregnancy.	0.710
10. I perform vaginal douching after urination.	0.843
11. I perform vaginal douching after a bowel movement (defecation).	0.853
12. I perform vaginal douching to avoid smelling bad.	0.920
13. I perform vaginal douching to feel clean.	0.917
14. I perform vaginal douching to feel good.	0.891
15. I perform vaginal douching before religious worship (e.g., before prayer/salah).	0.894
16. I perform vaginal douching before a gynecological examination.	0.886
17. I perform vaginal douching after a gynecological examination.	0.895
Eigenvalue	12.222
Variance explained	71.894%

### Confirmatory factor analysis and complementary indices

**Figure 2:**
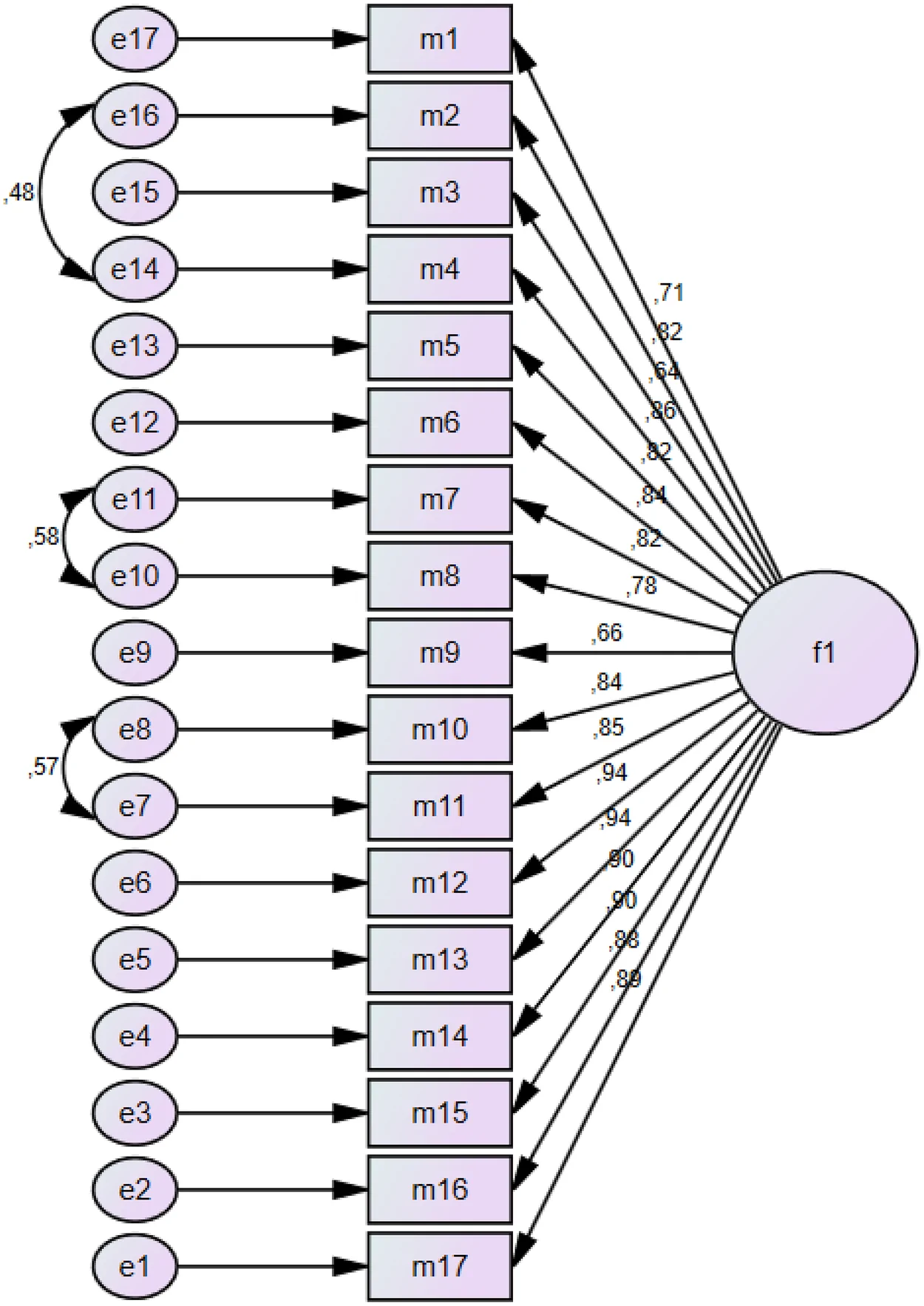
CFA path diagram for the one-factor model. The diagram includes three correlated residual pairs. These model modifications require theoretical justification and independent replication.

Standardized confirmatory factor analysis (CFA) loadings ranged from 0.642 to 0.937. Several incremental indices were at or slightly above 0.90 (CFI = 0.915, TLI = 0.900, IFI = 0.915, NFI = 0.903), but the absolute fit indices indicated substantial misfit (GFI = 0.786; RMSEA = 0.123, 90% CI = 0.116–0.131), and *χ*^2^/df was 4.428 (Table 5). On balance, the hypothesized one-factor model was not adequately supported. The path diagram also displayed three correlated residual pairs (Figure 2); these *post hoc* residual associations should be interpreted cautiously and require theoretical justification and replication.

**Table 5:** Confirmatory factor analysis Fit indices.

Index	Observed value	Interpretations
*χ*^2^/df	4.428	Marginal/poor; values below 3 are commonly preferred
GFI	0.786	Below conventional 0.90 benchmark
CFI	0.915	Minimally acceptable by a lenient 0.90 benchmark; below 0.95
TLI	0.900	Borderline
IFI	0.915	Minimally acceptable by a lenient 0.90 benchmark
NFI	0.903	Minimally acceptable by a lenient 0.90 benchmark
RMSEA	0.123	Poor fit

Fit benchmarks are interpretive guides rather than absolute rules. Overall fit should be judged across multiple indices and in relation to model specification.

CR was 0.975 and AVE was 0.694 (Table 6). These values indicate that the items share substantial variance with the modeled factor, but they do not override the inadequate global CFA fit or establish independent construct validity.

**Table 6:** Standardized CFA loadings and complementary reliability/validity indices.

Item/Index	Value
1. I perform vaginal douching before sexual intercourse	0.714
2. I perform vaginal douching after sexual intercourse.	0.818
3. I perform vaginal douching when I feel my menstrual period is about to start.	0.642
4. I perform vaginal douching after my menstrual period ends.	0.861
5. I perform vaginal douching when I have vaginal discharge.	0.823
6. I perform vaginal douching when I experience itching.	0.837
7. I perform vaginal douching to protect myself from sexually transmitted infections.	0.818
8. I perform vaginal douching to protect myself from cervical cancer.	0.775
9. I perform vaginal douching to prevent pregnancy.	0.664
10. I perform vaginal douching after urination.	0.836
11. I perform vaginal douching after a bowel movement (defecation).	0.850
12. I perform vaginal douching to avoid smelling bad.	0.937
13. I perform vaginal douching to feel clean.	0.936
14. I perform vaginal douching to feel good.	0.904
15. I perform vaginal douching before religious worship (e.g., before prayer/salah).	0.900
16. I perform vaginal douching before a gynecological examination.	0.884
17. I perform vaginal douching after a gynecological examination.	0.894
Composite reliability (CR)	0.975
Average variance extracted (AVE)	0.694

## Discussion

This study developed a 17-item instrument to quantify the frequency of vaginal douching across specific contexts. The revised construct definition resolves the previous inconsistency between “attitudes” and “practices”: the VDPS measures self-reported behavioral frequency, not evaluative attitudes, beliefs, or intentions.

The KMO and Bartlett's results indicated that data were suitable for factor analysis, and the EFA yielded a strong one-factor pattern with high factor loadings and 71.89% explained variance. However, EFA identifies a structure that best describes the observed sample; it does not, by itself, confirm that structure will reproduce in new data. Best-practice guidance recommends testing dimensionality in an independent sample or at a separate time point whenever possible (10, 12).

The CFA results did not adequately support the one-factor model. In particular, RMSEA of 0.123 and GFI of 0.786 indicated poor absolute fit, while CFI, TLI, IFI, and NFI were only minimally acceptable or borderline. The discrepancy between EFA and CFA may reflect local item dependence, redundant wording, a more complex latent structure, or sample-specific effects. The correlated residuals shown in the model further suggest shared content not fully explained by a single general factor. Accordingly, current findings should be described as preliminary rather than as definitive evidence of construct validity.

EFA and CFA were both conducted in the same sample. This approach can be used as an exploratory model checking exercise when resources are limited, but it offers weaker validation than a design using separate development and validation samples. The CFA therefore cannot be regarded as independent confirmation. Future studies should test the revised item set in a new sample, consider split-sample or cross-validation procedures when the sample permits, and evaluate alternative structures using theoretically justified models and contemporary factor-retention methods.

Internal consistency was very high. Cronbach's alpha of 0.97, CR of 0.975, and high corrected item-total correlations show that the items covary strongly. Nevertheless, coefficients approaching 1.00 can indicate redundant content.13 Items addressing closely related hygiene, cleanliness, and examination contexts should therefore be reviewed for conceptual overlap. Item reduction should be guided by content coverage, inter-item correlations, residual associations, and replication rather than by alpha alone.

The McDonald's omega coefficient also supported very high internal consistency. Nevertheless, the convergence of Cronbach's alpha, omega, CR, and high item-total correlations reinforces the need to evaluate possible item redundancy in future item-reduction studies.

The two-week test–retest correlation of 0.92 suggests rank-order stability, but the subsample of 31 participants was small and a correlation does not directly quantify absolute agreement. Future studies should use a larger prespecified subsample and report an intraclass correlation coefficient with a confidence interval.

Additional validity evidence remains necessary. No established instrument measuring the same behavioral construct was available for concurrent or convergent validation, and external criterion variables were not administered. Future studies could formulate *a priori* hypotheses relating VDPS scores to independently assessed douching frequency, genital hygiene behaviors, or relevant clinical indicators. Measurement invariance across marital status, age, geography, and cultural groups should be evaluated only after a stable factor structure is established.

The intended population in this initial study was married women attending family health centers in one region. The findings cannot be generalized to all women, particularly unmarried but sexually active women, adolescents, or women from other cultural and geographical settings. Until broader validation is completed, the VDPS should not be used as a diagnostic tool or to make individual clinical decisions. It may be used cautiously as a preliminary research measure, with scores interpreted continuously and with explicit acknowledgment of the unresolved factor-structure limitations.

## Conclusion

The 17-item Vaginal Douching Practice Scale (VDPS) demonstrated strong internal consistency, test–retest stability, and promising exploratory factor results. However, the CFA did not provide adequate support for the proposed one-factor model, and EFA and CFA were conducted in the same sample. The instrument should therefore be considered a preliminary measure rather than a fully validated scale. Item refinement, evaluation of redundancy, and independent validation in larger and more diverse populations are required before routine research or clinical use.

### Limitations

This study used convenience sampling and included only married women from one geographical region. EFA and CFA were performed in the same dataset, preventing independent confirmation. The CFA showed inadequate global fit, and *post hoc* correlated residuals require replication. Test–retest analysis included only 31 participants and used a correlation-based estimate. The very high internal consistency may reflect item redundancy. No external comparator or criterion measure was administered; therefore, convergent and criterion validity could not be evaluated, and discriminant validity between latent dimensions was not applicable to the one-factor model.

## Data Availability

The datasets generated and/or analyzed during the current study are available from the corresponding author upon reasonable request.
